# Neighborhood socioeconomic status is associated with low diversity gut microbiomes and multi-drug resistant microorganism colonization

**DOI:** 10.1038/s41522-023-00430-3

**Published:** 2023-08-28

**Authors:** Ibrahim Zuniga-Chaves, Shoshannah Eggers, Ashley E. Kates, Nasia Safdar, Garret Suen, Kristen M. C. Malecki

**Affiliations:** 1 Department of Bacteriology, University of Wisconsin-Madison, Madison, WI USA; 2 Microbiology Doctoral Training Program, University of Wisconsin-Madison, Madison, WI USA; 3 Department of Environmental Medicine and Public Health, Icahn School of Medicine at Mount Sinai, New York City, NY USA; 4 Department of Epidemiology, University of Iowa College of Public Health, Iowa City, IA USA; 5 Division of Infectious Disease, Department of Medicine, School of Medicine and Public Health, University of Wisconsin-Madison, Madison, WI USA; 6 Department of Population Health Sciences, School of Medicine and Public Health, University of Wisconsin-Madison, Madison, WI USA; 7 Environmental and Occupational Health Sciences, School of Public Health, University of Chicago Illinois, IL Chicago, USA

**Keywords:** Microbial ecology, Clinical microbiology, Policy and public health in microbiology, Microbiota

## Abstract

Social disparities continue to limit universal access to health care, directly impacting both lifespan and quality of life. Concomitantly, the gut microbiome has been associated with downstream health outcomes including the global rise in antibiotic resistance. However, limited evidence exists examining socioeconomic status (SES) associations with gut microbiome composition. To address this, we collected information on the community-level SES, gut microbiota, and other individual cofactors including colonization by multidrug-resistant organisms (MDROs) in an adult cohort from Wisconsin, USA. We found an association between SES and microbial composition that is mediated by food insecurity. Additionally, we observed a higher prevalence of MDROs isolated from individuals with low diversity microbiomes and low neighborhood SES. Our integrated population-based study considers how the interplay of several social and economic factors combine to influence gut microbial composition while providing a framework for developing future interventions to help mitigate the SES health gap.

## Introduction

Social inequality continues to grow in the USA as the income gap between the richest and the poorest Americans expands. In addition to political, economic, and social concerns related to rising economic inequality, there is also growing evidence linking income inequality to health disparities^1^. Widespread and notable disparities in health outcomes exist, in part, due to differences in socioeconomic status (SES)^2^. SES is shaped by both neighborhood context and individual level factors. Numerous individual-level factors link SES to adverse health outcomes including access to health care and personal behaviors^3–5^. However, neighborhood and health research has found that neighborhood-level SES can also predict health, even after accounting for these individual-level factors^6^. We know that individual SES is highly correlated with adverse environmental conditions, quality of housing, and lower social environment^7^. It is also now known that the accumulation of these factors, which results in low SES, also contributes to biological changes that are embedded across the life course, including altered composition and function of the human gut microbiome (HGM)^8,9^.

The HGM is the collection of all the microorganisms that regularly inhabits the human gastrointestinal tract. This community provides vital functions related to human health and disease^10^. Individuals have unique microbiomes, and their composition is determined by host genetics, diet, geographic location, environmental exposures, antibiotic usage, medical history, and overall activity^11–13^. Large changes to the “normal” microbiome can lead to a state of dysbiosis, commonly defined as an imbalance in the beneficial and non-beneficial organisms within the gut and is often associated with unfavorable conditions of health^14^. The development of current sequencing technologies has allowed researchers to discover and describe how changes in the HGM are associated with many health conditions including obesity, diabetes, inflammatory bowel disease, cancer, heart conditions, and neurological disorders^15–17^. Importantly, these chronic diseases have also been linked to SES, and although SES does not directly impact health status, it is a useful indicator of exposures^18–20^ that may lead to more severe deleterious health outcomes^21^.

SES in this context can shape individual-level access to resources including consistent access to nutrient-rich food. Food insecurity in the United States is a persistent and ongoing health crisis that is often exacerbated by changing and unstable economic conditions. In Wisconsin, food insecurity is prevalent across both urban and rural communities^22^. Food insecurity has also been linked with numerous adverse outcomes including cardiovascular disease and more recently, changes in gut microbial composition^23^.

Among the most acute changes that can occur as a result of altered gut microbial composition is antibiotic resistance. Recently, the WHO identified antibiotic resistance as a global health crisis. Antibiotic-resistant organisms make recovery from acute infections problematic and are most common among individuals who regularly seek health care, are older, or have a high prevalence of chronic health conditions such as obesity and diabetes^24^. The presence of antibiotic resistance, in turn, makes individuals more vulnerable to uncontrolled infection and mortality. More recently, the human gut microbiome and environmental determinants have also been shown to play an important role in driving antibiotic resistance^25^.

Previous work examining neighborhood-level socioeconomic conditions and the HGM^26–28^ revealed that numerous associations exist, but they did not identify how the interconnections of these factors might be influencing the observed associations. Furthermore, there is also a need to better understand the health implications of gut dysbiosis, with a primary concern being the prevalence of antibiotic-resistant bacteria^29,30^. Here, we address these knowledge gaps by leveraging the Survey of the Health of Wisconsin^31^ and its ancillary Wisconsin Microbiome Study^32^, which collects epidemiologic data and biological samples throughout urban and rural locations across the state of Wisconsin, USA with the goal of evaluating the overall health of the population. We hypothesize that individuals residing in lower-resourced communities will have different HGM compositions and that these associations are driven by both community and individual-level differences in SES, HGM composition and MDRO prevalence. We test this by developing analyses that examine: (1) the association between neighborhood-level SES and individual HGM composition; (2) the possible mediators of that association; and (3) the potential relationship between neighborhood-level economic hardship, HGM composition, and antibiotic-resistant bacteria colonization as a downstream health outcome.

## Results

### Study population and microbiome analysis

Descriptive analytics for the main variables are shown in Table 1 and a list of all variables included in our analysis is presented in Supplementary Table 1. A total of 721 individuals had complete information for both EHI and the microbiome and were included in the study. The average age for all groups was similar, ranging in the 50s, with females recruited more than males (Table 1). For the 50th percentile EHI groups, the annual income average was $57,000 for the high EHI and $78,000 for the low EHI groups. For the 85th percentile groups, the average was $42,000 for the high EHI and $72,000 for the low EHI groups.

**Table 1:** Distribution of demographics and potential covariates by EHI grouping from the microbiome study sample of the Survey of the Health of Wisconsin, 2016–2017.

	50th percentile EHI		85th percentile EHI	
	High EHI (*N* = 364)	Low EHI (*N* = 358)	High EHI (*N* = 106)	Low EHI (*N* = 616)
EHI score				
mean (sd)	3276.59 ± 653.31	1204.44 ± 665.68	4116.96 ± 195.82	1927.71 ± 1028.56
Age				
mean (sd)	53.95 ± 16.08	55.67 ± 16.23	50.05 ± 15.00	55.62 ± 16.23
Gender				
F	213 (59)	203 (57)	67 (63)	349 (57)
M	151 (41)	155 (43)	39 (37)	267 (43)
BMI				
mean (sd)	31.90 ± 8.05	29.58 ± 7.07	31.58 ± 8.37	30.61 ± 7.53
Food insecurity				
Yes	115 (32)	52 (15)	54 (51)	113 (18)
No	245 (67)	300 (84)	51 (48)	494 (80)
Missing	4 (1)	6 (2)	1 (1)	9 (1)
Antibiotic usage				
Yes	116 (32)	120 (34)	38 (36)	198 (32)
No	224 (62)	208 (58)	63 (59)	369 (60)
Missing	24 (7)	30 (8)	5 (5)	49 (8)
HH median income				
mean (sd)	56,990.08 ± 45,073.20	77,934.47 ± 52,615.93	42,139.42 ± 37,112.76	71,816.67 ± 50,726.69
College completed				
College complete	92 (25)	171 (48)	23 (22)	240 (39)
College incomplete	271 (75)	187 (52)	83 (78)	375 (61)
Added sugar intake (cups eq)				
mean (sd)	21.30 ± 33.19	12.75 ± 12.30	33.15 ± 50.26	14.12 ± 15.92
Medicaid insurance				
Yes	55 (15)	27 (8)	24 (23)	58 (9)
No	181 (50)	210 (59)	48 (45)	343 (56)
Missing	128 (35)	121 (34)	34 (32)	215 (35)

^a^Numbers inside brackets correspond to percentages unless stated otherwise.

For our microbiome analysis, sequencing yielded a total of 26,811,444 reads, with an average of 36,508 ± 1094 reads per sample that clustered into 6606 different amplicon sequence variants (ASVs) classified to 395 genera, 134 families, and 19 phyla. The distribution of the taxonomy at the phylum level was similar across all samples and was dominated by Firmicutes, Bacteroidota, Actinobacteriota, Verrucomicrobia, and Proteobacteria.

A total of 204 individuals had at least one isolated MDRO. Positive cultures for Methicillin-resistant *S. aureus* were found in 21 individuals, 129 were positive for fluoroquinolone-resistant Gram-negative bacilli, 36 for vancomycin-resistant *Enterococci* and 43 for *C. difficile*.

### Neighborhood EHI is associated with changes in the gut microbiome composition

**Fig. 1:**
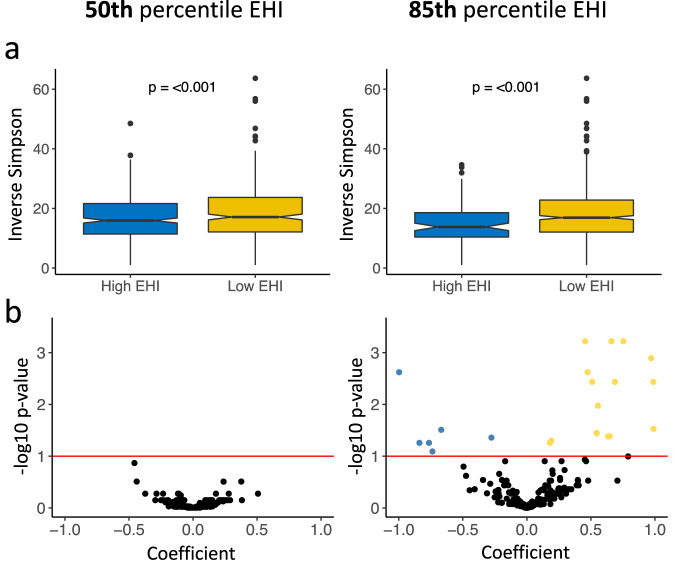
Neighborhood socioeconomic status is associated with changes in gut microbiome composition. **a** Box plots showing the comparison of the alpha diversity scores among gut microbiotas grouped by EHI scores (50th percentile EHI compares the top 50% (High EHI) against the bottom 50% of the population (Low EHI); 85th percentile EHI compares the top 15% (High EHI) against the bottom 85% of the population (Low EHI)). The notch shows the median of each group and significant *p* values for the simple linear models of EHI with the Inverse Simpson’s index are shown for each group. **b** ANCOM-BC log-linear model to determine genera that are differentially abundant according to EHI grouping. The y axis shows the negative logarithm of the adjusted *p* value while the x axis shows the correlation coefficient with the low EHI group as reference. Every point corresponds to a different genus and those above the red line had a *p* value adjusted for multiple comparisons (<0.1). Blue dots represent genera that are more abundant in the high EHI group and yellow points are more abundant in the low EHI group.

As the first step in our analysis, we modeled alpha diversity against neighborhood SES score and EHI. Within the study sample, the mean, median and range in EHI scores were 2248, 2311 and 26 to 4434 respectively. The observed richness, Shannon’s, and Inverse Simpson’s diversity metrics were all inversely proportional to the overall continuous EHI score (Supplementary Fig. 1). We decided to focus on Inverse Simpson to model our data as it is a robust measure of diversity that considers both bacterial richness and evenness. Fig. 1a shows the association of diversity to EHI dichotomized at the 50th and 85th percentiles. In both comparisons, corroborating our findings in the continuous EHI score model, individuals in the higher score category had significantly less diverse gut microbiomes (50th and 85th percentile EHI, *P* < 0.001).

We then evaluated changes in the taxonomic abundance between groups using ANCOM-BC to model the abundance of each genus identified in the samples. Fig. 1b displays taxa that were considered differentially abundant (adjusted *p* < 0.1 corrected with FDR) in both EHI groupings. When considering the 50th percentile EHI group, none of the genera showed a difference between groups. In contrast, the 85th percentile EHI comparison revealed 22 genera that were differentially abundant among the two groups, suggesting that differences at the taxonomy level are observed only in the most vulnerable sub-population. This finding was confirmed using the Bray-Curtis metric via PCoA plots (Supplementary Fig. 2). To corroborate the results identified by ANCOM-BC, the abundances of the 22 taxa were modeled with zero-inflated Poisson distribution (Supplementary Table 2) testing for both abundance (as counts) and presence/absence (zero inflation model). From both analyses, 6 genera were significantly more abundant in the 85th percentile EHI: 2 in the Actinobacteria and 4 in the Firmicutes. In contrast, 16 genera were more abundant in the Low EHI group: 3 in the Actinobacteria, 12 from the Firmicutes, and 1 from the Verrucomicrobia. Out of the differentially abundant taxa, only two genera had a relative abundance >3%: *Bifidobacterium* (3.4% below 85th percentile/ 5.9% 85th percentile EHI) and *Akkermansia* (3.7% below 85th percentile / 1.9% 85th percentile EHI).

### Food insecurity mediates a decrease in alpha diversity in high-EHI individuals

**Fig. 2:**
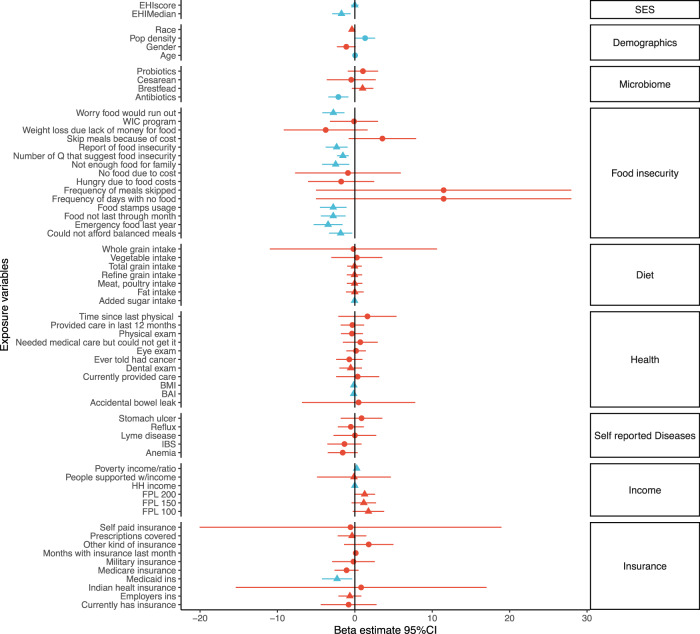
Estimated changes in alpha diversity by potential intermediate variables. The x-axis shows the 95% confidence intervals of each regression coefficient calculated with inverse Simpson as the dependent variable . The y-axis shows all possible intermediate variables grouped by categories. Blue lines indicate that the *p* value for the regression was <0.05 while red lines indicate a *p* value > 0.05. Lines with a triangle indicate variables with a statistically significant *p* regression with EHI as the dependent variable, whereas circles indicate not significant regressions.

**Fig. 3:**
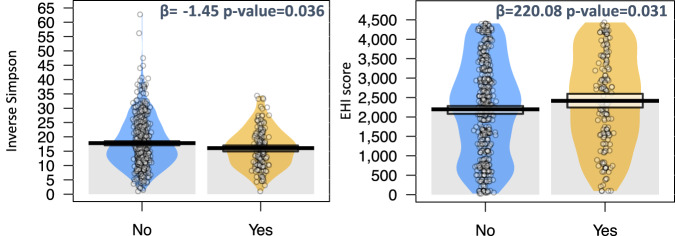
Association of MDRO prevalence with both EHI and alpha diversity. Violin plots showing the inverse Simpson’s diversity index and EHI scores based on the detection of MDRO isolates (yes vs no). Beta coefficients and *p* values of the linear regression are shown for each plot. The linear regressions were adjusted for antibiotic intake in each case.

Having established an association between high EHI and low diversity, we evaluated the association with other variables that may be intermediate. A total of 64 variables were tested, divided into 9 broad categories (Fig. 2). Of these, food insecurity was the one most associated with changes in diversity, with more than half being significantly associated with both lower diversity and higher EHI (Fig. 3). Moreover, a high household income, living in a rural community, and older age are associated with higher diversity and were the only variables in the demographic category that were significant. The use of antibiotics significantly decrease diversity, as expected, but was not associated with EHI. BMI and body adiposity index were the only health metrics associated with a decrease in diversity and higher EHI. Finally, none of the self-reported diseases were significantly associated with changes in diversity.

We then decided to build an adjusted model with all the variables that were associated with both EHI and diversity plus antibiotic usage. The final adjusted model, including EHI block, antibiotic usage, food insecurity, and BMI is shown in Table 2 for both grouping categories. Furthermore, mediation analysis was performed for both groupings of EHI to evaluate the variables identified as potential mediators in the adjusted model (Supplementary Table 3). For models evaluating EHI dichotomized at the 50th percentile, both food insecurity and BMI were significant mediators when only antibiotic usage was included as a covariate (*P* < 0.05, and significant bootstrap values). However, when other variables (BMI or food insecurity) were included, the indirect effect explained by the mediator decreased (BMI: *p* = 0.10, food insecurity: *p* = 0.058). For the 85th percentile EHI groups, food insecurity had a larger indirect effect (50th = 0.32 vs 85th = 0.56; *P* > 0.05), but BMI was not a significant mediator, as the indirect effect shrank to zero when both antibiotics and food insecurity were included as covariates. These results suggest that mediation by BMI does not apply to the most vulnerable population, and food insecurity explains most of the mediation captured by the model.

**Table 2:** Adjusted model for EHI association with alpha diversity.

	50th Percentile EHI		85th Percentile EHI	
	Estimate	*P* value	Estimate	*P* value
EHI score				
High EHI	Reference			
Low EHI	1.29	0.044	2.28	0.013
BMI				
Continous	−0.084	0.043	−0.095	0.023
Food insecurity				
Yes	Reference			
No	1.60	0.037	1.34	0.086
Antibiotic usage				
Yes	−1.80	0.0081	−1.74	0.010
No	Reference			
Missing	−2.76	0.034	−2.78	0.032

### The prevalence of MDROs is linked to both low alpha diversity and high EHI

We used the prevalence of MDRO isolates as an empirical indicator of participant health, rather than using other health outcomes that relied on self-reported data. We plotted the total number of MDRO isolates for all body sites with the HGM alpha diversity and the EHI score. Moreover, we modeled this association and adjusted it for antibiotic usage. As shown in Fig. 3, the prevalence of MDROs was negatively associated with HGM alpha diversity, indicating that participants were more likely to be colonized by MDROs if they had a less diverse gut microbiome. The same pattern was observed when considering the number of MDRO isolates per person, as shown in Supplementary Fig. 2A. Moreover, higher EHI was also associated with MDRO colonization (Fig. 3) and with a greater number of MDROs (Supplementary Fig. 2a). Finally, when we analyzed the number of MDRO isolates by EHI group, individuals in the 85th percentile were found to have a significantly higher number of isolates (Supplementary Fig. 2b). Hence, our MDRO data suggests that the more economic hardship experienced in a person’s neighborhood, the higher the probability of hosting one or more of these pathogens.

## Discussion

In this study, we provide evidence that individuals in low SES neighborhoods have lower gut microbiome diversity, which may play a role in shaping numerous chronic conditions and have an important relationship with antibiotic resistance. While SES, food insecurity, BMI and MDROs have been associated with changes in the gut microbiome in previous studies^2,33,34^, to our knowledge, this is the first report that provides evidence of how the interactions of numerous social and economic factors combine to influence gut microbial composition and diversity.

Neighborhood contextual socio-economic status is associated with higher levels of individual hardship and we sought to understand how these various determinants of SES shape gut health and downstream impacts. This idea has gained traction, as recent reviews suggest that social status, as part of the exposome, can lead to differential composition of the gut microbiome^30^. Among the numerous individual level metrics examined, including income, education, self-reported diseases, insurance information and diet, the most significant changes in gut microbial composition on an individual level were associated with food insecurity. Additionally, the results of our work using a neighborhood score were supported by other individual variables, indicating that other members of the community outside our cohort might be at risk. In a recent review, Robinson and colleagues considered the human microbiome in the context of social inequity^35^ and identified 20 important factors they believe contribute to social inequality, including the effect of food insecurity, on the prevalence of infectious disease. Our findings provide the first empirical evidence to support this assertion, highlighting the importance of food insecurity on human health at a community level.

In general, most microbiome-related diseases are characterized by a decrease in the overall number and distribution of microbes in the gut^36^, while a reduction in diversity may be indicative of increased disease risk^37,38^. Thus, our finding of a negative association between SES and diversity, reinforced by our MDRO data, supports the hypothesis that economic hardship could have a deleterious effect on the health of the microbiome, and may result in increased disease risk. Similarly, experimental evidence demonstrating that certain groups of gut bacteria can be beneficial to the host has been reported. For example, members of the genus *Akkermansia*, which we found to be abundant in the high SES group, promote mucus production, decrease inflammation and are associated with a reduced risk of obesity, Type 2 diabetes, IBD, and different types of cancer in mice^39,40^. Our finding that members of the families Lachnospiraceae (*Eubacterium* and *Frisingicoccus*) and Ruminococcaceae decreased in the high EHI groups may also be beneficial as these bacteria are known fiber degraders important in the modulation of the immune system and enhance function of enterocytes and the gut barrier^36^, which could be explained by the high added-sugar, low fiber diets that low SES (high EHI) families are subjected to in the USA. Finally, the genus *Bifidobacterium* was found to be more abundant in high EHI populations, despite being considered a beneficial microbe and a known probiotic^41^. Members of the *Bifidobacterium* are one of the first colonizers in the gut at birth^42^ and we hypothesize that our observation is a consequence of *Bifidobacterium* colonizing a less diverse gut microbiome, similar to what is encountered at birth, as described in other studies^43^. Further research is needed to elucidate the mechanism of association between these microbes, SES, and their impact on downstream health.

One of the main challenges in understanding the relationship between SES and the HGM is in identifying specific exposure mechanisms that underpin these associations. Access to adequate amounts of food is a persistent problem in the USA, where 11% of households are food insecure^34^. Food insecurity, food deprivation, and malnutrition are deleterious to both the human gut and the HGM^44^ by disrupting adequate gut barrier function, which can lead to dysbiosis of the HGM and a higher prevalence of disease and pathogen colonization. Beyond food deprivation, food insecurity also limits access to nutrient-rich food. High-fat and low-fiber diets are common among individuals with low SES in high-income countries where they are encouraged to purchase and consume foods that have the highest calories per dollar^19^. Even in food-insecure populations, nutrient-rich diets seem to influence the gut microbiome, indicating that both food availability and quality are potential modifiers of the intestinal microbial community^23^. Excessive amounts of refined sugars and fat found in these calorie-dense foods can lead to additional dysfunction in gut enterocytes and increase the risk for cardiovascular disease^45^. This type of diet can also lead to fat accumulation and obesity, suggesting a possible explanation for BMI as a mediator, although we note that BMI was not a significant mediator in our cohort. In high-income countries, obesity is associated with low SES, but the opposite is true for low-income countries where obesity is associated with high SES^46^. A study of SES and the HGM in a Chinese cohort found associations between high SES, increased BMI and a higher prevalence of metabolic syndrome^28^. Thus, the mediation observed with BMI is likely dependent on geographic location, diet, and cultural differences, rather than food insecurity, which is widely associated with low SES.

We were also able to leverage MDRO prevalence to identify an association between SES and altered microbiome composition. MDROs are usually found in hospital settings where there is constant use of antibiotics. However, they are now appearing in rural and urban settings outside of healthcare facilities^47,48^. Colonization by MDROs does not equate to disease, but it does increase risk of infection^49^ and dissemination in high EHI communities due to low SES and crowded housing^50^. Also, they have the potential to provoke an active infection in the event that an individual’s immune system is depressed by a secondary disease or malnutrition, which is likely more prevalent in high EHI neighborhoods^51^. Hosting a diverse microbiome is known to provide protection against foreign and potentially pathogenic microbes, including MDROs. The higher prevalence of MDROs in individuals with low SES may be due to a lack of competitive inhibition by a diverse microbiome^52^. Additionally, chronic stress, inflammation, and poor nutrition, which are common in individuals with low SES, may provide additional pathways for MDRO colonization in EHI communities.

Although our results provide new insights into the HGM and MDRO colonization in association with both community and individual level SES, we acknowledge that our study has limitations. For example, our study recorded the use of antibiotics as a binary outcome, and we recognize that the dose, frequency, and type of antibiotic treatment could influence our results as these factors are known to have a direct impact on the gut microbiome. However, given the goals of this study, we decided to consider broad and widespread effects within this population. We further note that considering only those individuals who provided specific antibiotic usage data would have greatly reduced our sample size. Moreover, although cross-sectional studies allow only for the identification of associations, our study incorporates a wealth of epidemiological data, which is difficult to collect in a time series, given the challenges in gathering a large cohort for a SES-controlled experiment. Finally, we note that the associations observed in our study are based on 16S rRNA amplicon sequencing, which only allowed us to evaluate bacterial community composition. The use of this technique does not allow for strain-level information of the microbes in the gut or their function. Alternative methods such as shotgun metagenomics could be used in the future to gain insights into the functional potential of the microbiome when exposed to different levels of EHI.

In this study, we provide evidence for the association of community-SES and changes in the HGM. We found that residing in low SES neighborhoods appears to negatively impact the diversity and composition of an individual’s gut microbiome, which could lead to lower competition, decreases in the abundance of commensals, and increase the risk for pathogen invasion. Individual factors including food insecurity and BMI, both support and provide possible explanations for this association. With the goal of achieving a universal healthy microbiome, we expect this work to not only provide a framework for future interventions but underscore the importance of further population studies with different cultural and geographic contexts to support our findings.

## Methods

### Study design and population

This study utilized extant data from the Survey of the Health of Wisconsin (SHOW)^31^ and its ancillary study, the Wisconsin Microbiome Study (WMS)^32^. SHOW is a statewide study that began in 2008 by enrolling participants across Wisconsin using census blocks. The main goal of the SHOW is to collect health exposure and outcome data addressing all major determinants of health, including healthcare access, social determinants, lifestyle, and behavioral factors. In 2016, the SHOW survey included standard data collection coupled with swabs of the skin, nose, and mouth and samples of stool and saliva as part of the WMS. A full description of the SHOW inclusion/exclusion criteria, collected data, as well as other ancillary projects such as the WMS, can be obtained at: https://show.wisc.edu. For this project, we included every individual age 18 or older that had completed the SHOW data collection and had stool samples available from the 2016 and 2017 collection periods.

### Inclusion and ethics statement

The studies involving human participants were reviewed and approved by the University of Wisconsin-Madison Institutional Review Board. The patients/participants provided their written informed consent to participate in this study.

### Biospecimen availability

All biospecimens associated with this study are available through the State Health of Wisconsin (SHOW) statewide-representative cohort at: https://show.wisc.edu/services/biospecimen/.

### Main exposure and covariates

To evaluate SES, we used the Economic Hardship Index (EHI) as a proxy for neighborhood SES. The EHI was developed by the Rockefeller Institute of Government and is derived from data from the 2000 United States census^53,54^. It is a block group level score that includes six indicators representing a neighborhood’s social and economic features. High EHI scores represent more hardship or burden in a neighborhood and correspond to lower SES. Indicators include: crowded housing (percentage of occupied housing with more than one person per room), poverty status (percentage of persons living below 100% federal poverty level), unemployment (percentage of persons over the age of 16 who are unemployed), education (percentage of persons over the age of 25 without a high school education), dependency (percentage of the population under 18 or over 64 years of age), and individual annual income (tertiles of < $20,000; $20,000–44900; and >$45000)^55^. To create the EHI composite scores, each indicator was characterized and summed. Census block groups within entire state of Wisconsin were ranked according to the counts in each category and assigned an overall score. Each SHOW participant household was geocoded to a street address and linked to their census block group level EHI score as a continuous metric.

To evaluate possible individual SES factors that may serve as confounders or meditating variables, we included additional information obtained from SHOW questionnaires and personal interview^56^. In summary, we included relevant demographic information regarding gender and age at the time of consent and the level of education and household income. Other health information, such as the use of any antibiotic treatments in the last year, self-reported diseases, insurance, and measures of body mass index were also included. Finally, we included diet-related data such as consumption of different food groups based on the food patters equivalents database^57^ and information on food security. Table 1 provides a detailed description of all variables included in this study.

### Microbiota analysis

A detailed description of sample collection, DNA extraction, and amplification has already been described elsewhere^32^. Briefly, DNA was extracted using phenol-chloroform and the V4 region of the 16S rRNA gene was amplified via polymerase chain reaction using V4-specific bacterial primer (515F: GTGCCAGCMGCCGCGGTAA and 806R: GGACTACHVGGGTWTCTAAT) followed by sequencing on an Illumina MiSeq at the University of Wisconsin Biotechnology Center. Negative controls were included during extraction and amplification and were sequenced.

The resulting raw sequences from the sequencer were processed using the software package QIIME 2 v2021.4^58^. Demultiplexed raw sequences were imported using the Casava 1.8 format and denoised using DADA2 v1.18.0^59^ (via qiime-dada2 plugin) to generate a feature table containing amplicon sequence variants (ASV). ASVs were aligned with MAFFT v7.475 and used to construct a phylogenetic tree using FastTree v2.1.1^60^. Taxonomy was assigned using the classify‐sklearn naive Bayes taxonomy classifier v0.24.1^61^ (via qiime-feature classifier plugin) and the Silva_138 database for 16S rRNA genes^62^. Both feature and taxonomy tables, together with the phylogenetic tree, were imported into R as a phyloseq object^63^ for further analysis. Contaminants were eliminated based on the prevalence of ASVs in the negative controls using the package Decontam^64^ and by removing all ASVs classified as belonging to Eukaryotic, chloroplast, mitochondrial or unassigned taxa. Samples with less than 5000 reads were removed and samples were subsampled to an even depth of 8396. Alpha diversity metrics, including Shannon’s diversity^65^, inverse Simpson’s^66^, and total observed ASVs were calculated using phyloseq.

### Multidrug-resistant organism (MDRO) prevalence

Detailed methods for the isolation of (MDROs) have been published elsewhere^32^. Briefly, swabs, saliva, and stool samples were screened for the presence of 4 multidrug-resistant pathogens: methicillin-resistant *Staphylococcus aureus*, vancomycin-resistant enterococci, *Clostridioides difficile* and fluoroquinolone-resistant gram-negative bacilli. Subsamples from each sample were inoculated on selective media with antibiotics and checked for growth. Matching colonies were subsampled onto blood agar plates for confirmatory identification using biochemistry assays and further verified by sequencing the 16S rRNA gene. Further confirmation for antibiotic-resistant phenotypes was performed using Kirby- Bauer disc diffusion and *E* test strips.

### Statistical analysis

To facilitate the visualization and analysis of the data, individuals were clustered into blocks based on their EHI scores using two methods: (1) dichotomized at the median EHI, and (2) dichotomized at the 85th percentile EHI. The first method was used to evaluate the effect of residing in neighborhoods within the top half of the economic hardship index and the second was used to investigate a subset of the population residing in neighborhoods with exceptionally low SES. Simple linear regressions were calculated for every variable, included against the inverse Simpson’s index to evaluate associations with alpha diversity. Variables that showed a significant association with changes in alpha diversity were also modeled with EHI scores. Variables that showed associations with both SES and EHI were included to build an adjusted model together with antibiotic usage. To obtain the most parsimonious model, a reverse selection was used to remove covariates with *p* value > 0.1. To evaluate mediation, the simple mediation extension in the R package MeMoBootR^67^ was used. Both Sobel and permutation tests were performed to evaluate the significance of the results obtained in every mediation. Mediation analyses were adjusted for the same variables as the final linear regression models. Differential taxonomic abundance was obtained using the ANCOM-BC package in R, using default parameters for cross-sectional data^68^. Identified taxa were confirmed with Zero-Inflated Poisson modeling adjusted for antibiotic use^69^.

### Reporting summary

Further information on research design is available in the Nature Research Reporting Summary linked to this article.

### Supplementary information

Supplementary material

Reporting Summary

## Data Availability

All sequences associated with this study have been deposited into the National Center for Biotechnological Information’s Short Read Archive and are available under BioProject ID PRJNA999362.

## Code availability

All code that supports the findings of this study are available from the corresponding author upon request.
