# Supplementary material for: Neighborhood socioeconomic status is associated with low diversity gut microbiomes and multi-drug resistant microorganism colonization
Source: NPJ Biofilms Microbiomes. 2023 Aug 28;9:61. doi: 10.1038/s41522-023-00430-3 (PMC10462741; doi:10.1038/s41522-023-00430-3)
Supplement: Supplementary file 1 — Supplementary material [file 41522_2023_430_MOESM1_ESM.pdf]

Supplementary Table 1. List of all epidemiological variables included from SHOW

Variable	SHOW_code	Description
EHIscore	EHI_CBG_RANK_ACS2015	Economic hardship index by block
EHI 50th percentile	avg_EHI	Economic hardship index by block clustered by the 50th percentile
College_education	DMQ010CAT_onlycollege	Person attended college or no.
Antibiotics	HMI070	Antibiotic treatment in the last year
Probiotics	HMI080	Probiotics used in the last year
Cesarean	HMI010	Where you born by c-section?
Brestfead	HMI020	Where you breastfeded?
Gender	GENDER	Binary gender
Race	RACE_ETHNICITY	White or not white
Age	AGE	Age at the time of consent
Pop density	CENSUS_UAUC_2CAT	Do you live in an Urban or Rural area.
HH income	INCOME_HH_MID	Yearly household income
People supported w/income	INQ201	Number of people supprted with household income
FPL 100	POVERTY_100	Participant above of below the 100% Federal poverty level
FPL 150	POVERTY_150	Participant above of below the 150% Federal poverty level
FPL 200	POVERTY_200	Participant above of below the 200% Federal poverty level
Poverty income/ratio	POVERTY_IR	Poverty over income ratio
Medicaid ins	IUQ020_MEDICAID_R2	Are you enrolled in Medicaid insurance?
Months with insurance last month	IUQ010	Number of months with insurance as of last month
Prescriptions covered	IUQ030	Portion of prescription covered by insurace
Employers ins	IUQ020_EMPL	Are you enrolled with your employers insurance?
Self paid insurance	IUQ020_INDIV	Are you directly paying for your insurance.?
Medicare insurance	IUQ020_MEDICARE	Are you enrolled in Medicare insurance?
Military insurance	IUQ020_MIL_R2	Are you enrolled in Military insurance?
Indian healt insurance	IUQ020_IHS_R2	Are you enrolled in Indian Health insurance?
Other kind of insurance	IUQ020_OTHERIND	Are you enrolled in any other type insurance not included in the list?
Currently has insurance	CURRENT_INSURANCE	Do you currently have insurance
Ever told had cancer	HHQ480	Have you ever been told to have cancer?
Accidental bowel leak	INC037	Have you ever had a case of accidental bowel leak?
Time since last physical	IUQ220_Nrange	Approximate time since last medical physical consultation
Needed medical care but could not get it	IUQ260_R2	Have youn ever needed medical care but were not able to get it?
Physical exam	PSH050	What was the last time you got a physical exam ?
Eye exam	PSH070	What was the last time you got a eye exam ?
Dental exam	PSH080	What was the last time you got a dental exam ?
Currently provided care	CGQ020	Are you currently getting provided care
Provided care in last 12 months	CGQ010	Have you had any provided care in the last 12 months
Worry food would run out	FSQ002	Are you ever worried food would run out?
Food not last trough month	FSQ003	Are you ever worried food wound not last through the month?
Could not afford balance meals	FSQ004	Are you not able to afford balance meals ?
Number of Q that suggest food insecurity	FSQ_TOTAL3_R2	Number of questions anwsered yes that indicate food insecurity
Emergency food last year	FSQ151R2	Have you ever been told to have cancer?
Food stamps usage	FSQ170R2	Have you ever used food stamps
WIC program	FSQ162R2	Have you ever used the WIC program
Food insecurity	FSQ_ANYISSUE3_R2	If the participant anwser yes to any question related to food security
Not enough food for family	FSQ080	Do you ever worry about not having enough food for the family?
Skip meals because of cost	FSQ010	Do you ever skip meals because of cost?
Frequency of meals skiped	FSQ011	What is the frecuency of meals skipped because of cost?
Hungry due to food costs	FSQ013	Are you ever hungry because of food cost?
Weight loss due lack of money for food	FSQ014	Have you ever experience weight loss because of lack of money?
No food due to cost	FSQ020	Have you ever experience days with food shortage because of cost?
Frequency of days with no food	FSQ021	How often do you experiece food shortages because of cost?
Anemia	HHQ580_3	Have you ever been diagnosed with anemia?
IBS	HHQ580_19	Have you ever been diagnosed with inflamatory bowel disease?
Lyme disease	HHQ580_22	Have you ever been diagnosed with lyme disease?
Reflux	HHQ580_31	Have you ever been diagnosed with reflux?
Stomach ulcer	HHQ580_35	Have you ever been diagnosed with anemia?
Added sugar intake	FPED_ADD_SUGARS	Number of added sugars teaspoon equivalents consumed according to the Food Patterns Equivalents Database (FPED) (https://data.nal.usda.gov/dataset/food-patterns-equivalents-database-fped)
Vegetable intake	FPED_V_TOTAL	Number of vegetable cup equivalents consumed according to the Food Patterns Equivalents Database (FPED) (https://data.nal.usda.gov/dataset/food-patterns-equivalents-database-fped)
Refine grain intake	FPED_G_REFINED	Number of refine grain ounce equivalents consumed according to the Food Patterns Equivalents Database (FPED) (https://data.nal.usda.gov/dataset/food-patterns-equivalents-database-fped)
Total grain intake	FPED_G_TOTAL	Number of total grain ounce equivalents consumed according to the Food Patterns Equivalents Database (FPED) (https://data.nal.usda.gov/dataset/food-patterns-equivalents-database-fped)
Whole grain intake	FPED_G_WHOLE	Number of whole grain ounce equivalents consumed according to the Food Patterns Equivalents Database (FPED) (https://data.nal.usda.gov/dataset/food-patterns-equivalents-database-fped)
Meat, poultry intake	FPED_PF_MPS_TOTAL	Number of Meat, poultry, seafood protein food ounce equivalents consumed according to the Food Patterns Equivalents Database (FPED) (https://data.nal.usda.gov/dataset/food-patterns-equivalents-database-fped)
Fat intake	FPED_SOLID_FATS	Number of fat gram equivalents consumed according to the Food Patterns Equivalents Database (FPED) (https://data.nal.usda.gov/dataset/food-patterns-equivalents-database-fped)
BMI	ANT_BMI	Body mass index
BAI	ANT_BAI	Body adiposite index

Supplementary Table 2. Differences in the abundance of the identified genus in the 85th percentile EHI group comparing ANCOM-BC with zero-inflated Poisson (ZIP) models. The number and direction of the arrows indicate both the significance and EHI group in which the genus was more abundant.

Genus	Total rel. abundance 100%	ANCOMBC	ZIP abundance	ZIP presence/absence
Bifidobacterium	**3.78**	↑	↑↑↑	**ns**
Akkermansia	**3.44**	↓↓	↓↓↓	**ns**
Holdemanella	**0.42**	↑	↑↑↑	↑↑↑
Clostridia_UCG014	**0.81**	↓↓↓	↓↓↓	↓↓↓
Christensenellaceae_R7_group	**0.72**	↓↓↓	↓↓↓	↓↓↓
Catenibacterium	**0.43**	↑	↑↑↑	↑↑↑
Ruminococcaceae_uncultured	**0.32**	↓↓	↓↓↓	↓↓
*Eubacterium_ventriosum*_group	**0.25**	↓↓	↓↓↓	↓↓↓
NK4A214_group	**0.24**	↓↓	↓↓↓	↓↓
Megasphaera	**0.15**	↑↑↑	↑↑↑	↑↑↑
Acidaminococcus	**0.11**	↑↑	↑↑↑	↓↓↓
*Coriobacteriales_Incertae_Sedis*_uncultured	**0.09**	↓↓	↓↓↓	↓↓↓
Frisingicoccus	**0.09**	↓↓↓	↓↓↓	↓↓↓
Colidextribacter	**0.09**	↓↓↓	↓↓↓	↓↓↓
*Eubacterium_xylanophilum*_group	**0.07**	↓↓↓	↓↓↓	↓↓↓
UCG010	**0.06**	↓↓↓	↓↓↓	↓↓↓
Candidatus_*Stoquefichus*	**0.05**	↓↓↓	↓↓↓	↓↓↓
Parvibacter	**0.04**	↓↓	↓↓↓	↓↓↓
Peptococcaceae_uncultured	**0.02**	↓↓↓	**ns**	↓↓↓
Ruminiclostridium	**0.01**	↓↓	↓↓↓	↓↓

Coriobacteriales_uncultured	**0.01**	↓↓	↓↓↓	↓↓
Atopobium	**<0.01**	↑↑	↑↑↑	↑↑↑

Pvalue **ns** >0.1 ↑ 0.1-0.05 ↑↑ 0.05-0.01 ↑↑↑ <0.01 **Group** ↑ HighEHI ↓ LowEHI

Supplementary Table 3. Results of the mediation analysis for the association of EHI and alpha diversity. The values in column n correspond to the total number of participants included in the model. Food insecurity and BMI were the covariables chosen for our mediation analysis. Both models with and without covariables are shown. Significant mediation was corroborated by a Sobel test <0.05 and a Bootstrap CI that does not cross zero.

n	X	Y	Mediator	Covariates	Indirect effect	Sobel test	Bootstrap 95% confidence interval
702	50th Percentile EHI	InverseSimpson	Food insecurity	Antibiotics	0.32	0.028	(0.0614, 0.5792)
702	50th Percentile EHI	InverseSimpson	Food insecurity	Antibiotics, BMI	0.25	0.058	(0.0069, 0.4988)
702	50th Percentile EHI	InverseSimpson	BMI	Antibiotics	0.22	0.047	(0.0021, 0.4346)
702	50th Percentile EHI	InverseSimpson	BMI	Antibiotics, Food insecurity	0.15	0.102	(-0.0324, 0.3281)
702	85th Percentile EHI	InverseSimpson	Food insecurity	Antibiotics	0.56	0.041	(0.0399, 1.0723)
702	85th Percentile EHI	InverseSimpson	Food insecurity	Antibiotics, BMI	0.44	0.096	(-0.0522, 0.9461)
702	85th Percentile EHI	InverseSimpson	BMI	Antibiotics	0.11	0.294	(-0.1144, 0.3209)
702	85th Percentile EHI	InverseSimpson	BMI	Antibiotics, Food insecurity	0	0.915	(-0.2190, 0.1979)

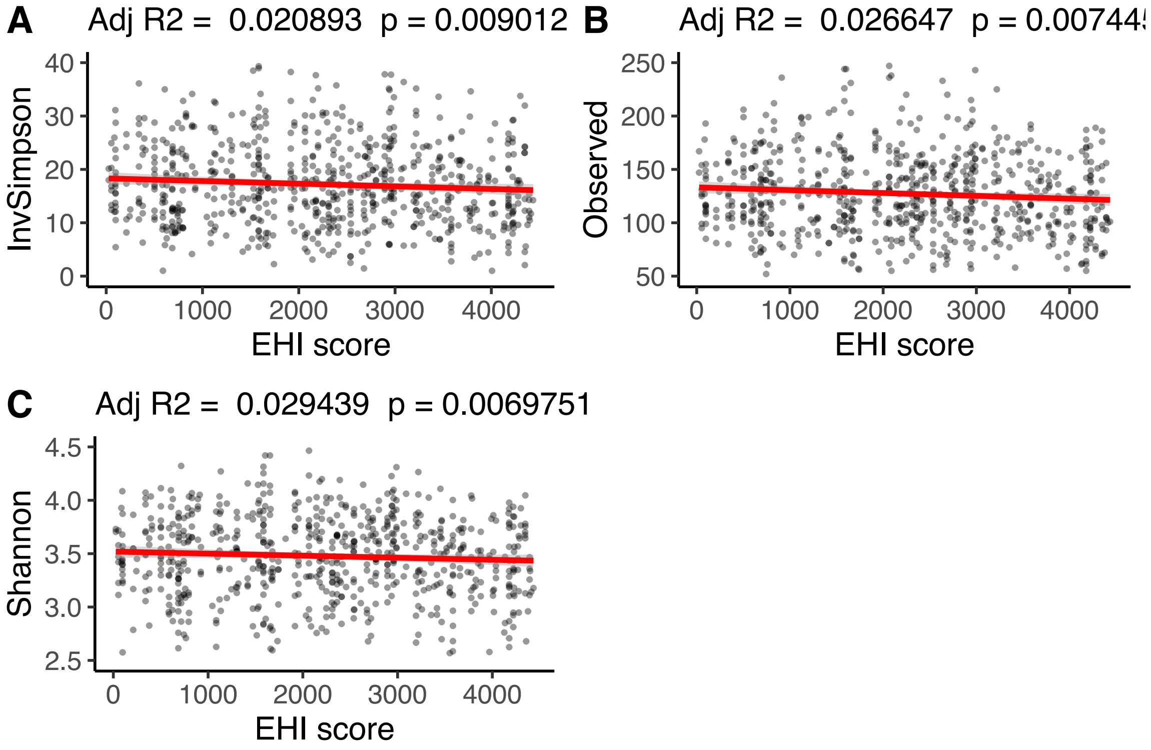

Supplementary Figure 1. Association of alpha diversity and EHI score. A) Inverse Simpson's, B) Observed ASVs and C) Shannon's diversity. The adjusted R2 and p values for the association are shown for each metric.

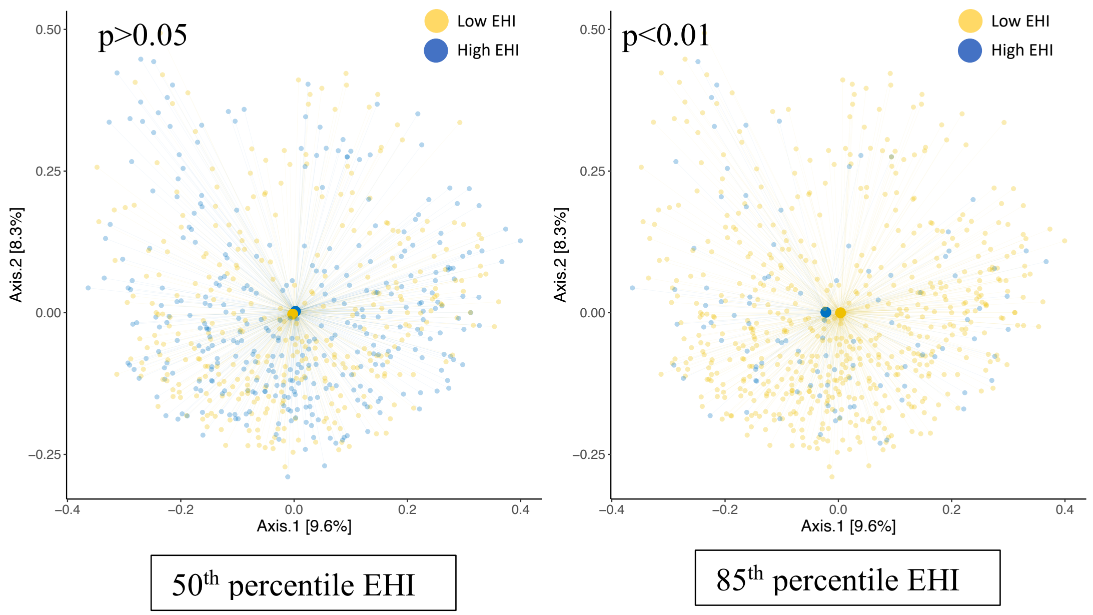

Supplementary Figure 2. Differences in microbial communities by EHI groups. Principal component analysis of the gut microbiome of all individuals included in the study. Distances were calculated using the Bray-Curtis dissimilarity index. Points are plotted based on EHI percentile groups. The centroids for each treatment group show overall differences between communities.

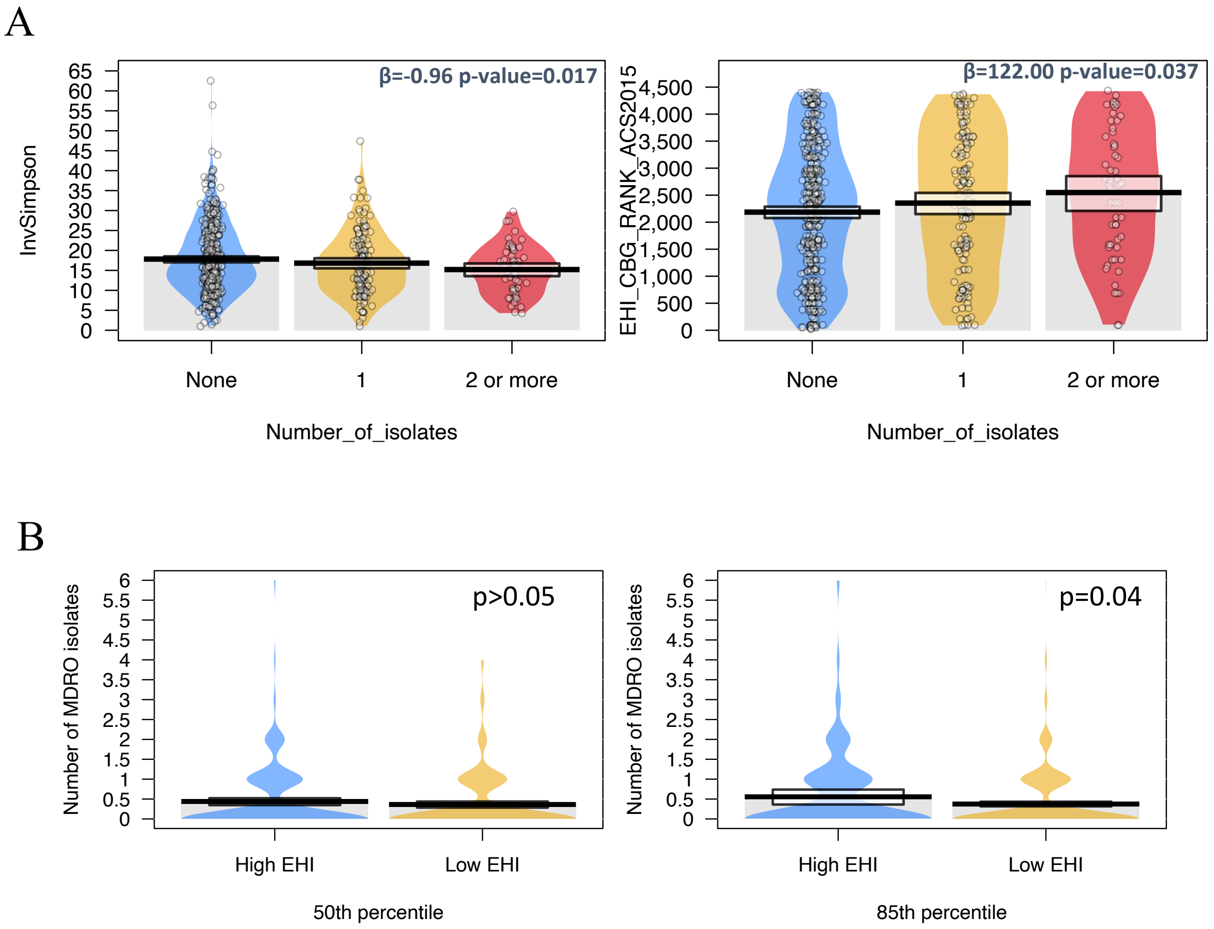

Supplementary Figure 3. Association of MDRO prevalence with both EHI and alpha diversity. A) Violin plots showing the inverse Simpson's diversity index and EHI scores based on the number of MDRO isolates. Beta coefficients and p-values of the linear regression are shown for each plot. The linear regressions were adjusted for antibiotic intake in each case. B) Differences in the number of MDRO isolates by EHI percentile group. P values indicate if significant differences exist using a Wilcoxon test.
