# Supplementary material for: Neighborhood socioeconomic status is associated with low diversity gut microbiomes and multi-drug resistant microorganism colonization
Source: NPJ Biofilms Microbiomes. 2023 Aug 28;9:61. doi: 10.1038/s41522-023-00430-3 (PMC10462741; doi:10.1038/s41522-023-00430-3)
Supplement: Supplementary file 2 — Reporting Summary [file 41522_2023_430_MOESM2_ESM.pdf]

nature portfolio

Corresponding author(s): Garret Suen & Kristen Malecki

Last updated by author(s): Aug 14, 2023

Reporting Summary

Nature Portfolio wishes to improve the reproducibility of the work that we publish. This form provides structure for consistency and transparency in reporting. For further information on Nature Portfolio policies, see our Editorial Policies and the Editorial Policy Checklist.

Statistics

For all statistical analyses, confirm that the following items are present in the figure legend, table legend, main text, or Methods section.

n/a	Confirmed	
☐	☒	The exact sample size (*n*) for each experimental group/condition, given as a discrete number and unit of measurement
☐	☒	A statement on whether measurements were taken from distinct samples or whether the same sample was measured repeatedly
☐	☒	The statistical test(s) used AND whether they are one- or two-sided<br>*Only common tests should be described solely by name; describe more complex techniques in the Methods section.*
☐	☒	A description of all covariates tested
☐	☒	A description of any assumptions or corrections, such as tests of normality and adjustment for multiple comparisons
☐	☒	A full description of the statistical parameters including central tendency (e.g. means) or other basic estimates (e.g. regression coefficient) AND variation (e.g. standard deviation) or associated estimates of uncertainty (e.g. confidence intervals)
☐	☒	For null hypothesis testing, the test statistic (e.g. *F*, *t*, *r*) with confidence intervals, effect sizes, degrees of freedom and *P* value noted<br>*Give P values as exact values whenever suitable.*
☒	☐	For Bayesian analysis, information on the choice of priors and Markov chain Monte Carlo settings
☐	☒	For hierarchical and complex designs, identification of the appropriate level for tests and full reporting of outcomes
☐	☒	Estimates of effect sizes (e.g. Cohen's *d*, Pearson's *r*), indicating how they were calculated

Our web collection on statistics for biologists contains articles on many of the points above.

Software and code

Policy information about availability of computer code

Data collection	N/A
Data analysis	N/A

For manuscripts utilizing custom algorithms or software that are central to the research but not yet described in published literature, software must be made available to editors and reviewers. We strongly encourage code deposition in a community repository (e.g. GitHub). See the Nature Portfolio guidelines for submitting code & software for further information.

Data

Policy information about availability of data

All manuscripts must include a data availability statement. This statement should provide the following information, where applicable:

- Accession codes, unique identifiers, or web links for publicly available datasets
- A description of any restrictions on data availability
- For clinical datasets or third party data, please ensure that the statement adheres to our policy

The data that support the findings of this study are available from the corresponding author upon reasonable request. The data were collected by the Wisconsin Microbiome Project, An Ancillary Study to the Survey of the Health of Wisconsin funded by the Wisconsin Partnership program and are not currently available as public use data at the time of manuscript preparation. All biospecimens associated with this study are available through the State Health of Wisconsin (SHOW) statewide-representative cohort at: https://show.wisc.edu/services/biospecimen/.

Research involving human participants, their data, or biological material

Policy information about studies with human participants or human data. See also policy information about sex, gender (identity/presentation), and sexual orientation and race, ethnicity and racism.

Reporting on sex and gender	Data used in this study considered only gender as a demographic descriptor as described in Malecki et al. (doi: 10.3389/fpubh.2022.818777). Gender was self-reported by each individual. All participants provided their written informed consent to participate in this study and to share individual-level data.
Reporting on race, ethnicity, or other socially relevant groupings	The study sample was randomly chose to represent the population of the state of Wisconsin, hence no race/ethnic groups were targeted. Respondents selected their race/ethnicity groups out of four options: White non-Hispanic, Black non-Hispanic, Hispanic and Other. All participants provided their written informed consent to participate in this study and to share individual-level data.
Population characteristics	The study used an existing dataset from the Survey of the Health of Wisconsin project and its ancillary project the Wisconsin Microbiome study. Includes an adult cohort (age >18 years old) of 721 individuals from the state of Wisconsin. A detailed summary of the population characteristics has already been describe by Eggers et al. (doi: 10.1136/bmjopen-2017-019450)
Recruitment	Recruitment of SHOW participants begins with in-person contact by study staff at the selected household address. Also, to ensure public awareness and increase participation, a public relations campaign was launched in communities six to eight weeks before recruitment in that location. Details on the recruitment process for SHOW are described in more detailed by Nieto et al. (doi:10.1186/1471-2458-10-785) and Malecki et al. (doi: 10.3389/fpubh.2022.818777)
Ethics oversight	University of Wisconsin Institutional Review Board

Note that full information on the approval of the study protocol must also be provided in the manuscript.

Field-specific reporting

Please select the one below that is the best fit for your research. If you are not sure, read the appropriate sections before making your selection.

☐ Life sciences ☐ Behavioural & social sciences ☒ Ecological, evolutionary & environmental sciences

For a reference copy of the document with all sections, see nature.com/documents/nr-reporting-summary-flat.pdf

Ecological, evolutionary & environmental sciences study design

All studies must disclose on these points even when the disclosure is negative.

Study description	This is a cross sectional study in an adult cohort from Wisconsin, USA to evaluate the the relation of Neighborhood SES and gut microbiome composition, identify mediators and link it to Multi drug Resistant Organisms.
Research sample	The study used an existing dataset from the Survey of the Health of Wisconsin (SHOW) project and its ancillary project the Wisconsin Microbiome Study as described in Eggers et al. (doi: 10.1136/bmjopen-2017-019450). Stool samples, survey data and cultured pathogens tested for multi-drug resistance were obtained from an adult cohort (age >18 years old) of 721 individuals from these projects.
Sampling strategy	Survey participants were selected from a random sample of households using a three-stage, probability-based cluster sampling approach to generate a sampling frame . A detailed description of the sampling procedure is available in Malecki et al. (doi: 10.3389/fpubh.2022.818777). Sample size calculations are described in Eggers et al. (doi: 10.1136/bmjopen-2017-019450). The sample size of over 700 subjects used in this study provided 80% power to detect a partial correlation (after adjustment for covariates) between the main variables and the primary outcome using a two-sided 2.5% level test.
Data collection	Data collection is divided into three major components: an in-home interview (Time 1); a self-administered questionnaire (Time 2); and a mobile exam center or fixed clinic visit that includes a physical exam, biospecimen collection, and more personal data collection (Time 3). An extensive description of the collection protocol for these components are available by Nieto et al. (doi: 10.1186/1471-2458-10-785). For the stool samples, participants self-collected at home using a collection kit provided by the project administrators as described by Eggers et al. (doi: 10.1136/bmjopen-2017-019450)
Timing and spatial scale	The samples used in this study correspond to the second phase of the State Health of Wisconsin study and the Wisconsin Microbiome project as describe in Malecki et al. (doi: 10.3389/fpubh.2022.818777) and Eggers et al. (doi: 10.1136/bmjopen-2017-019450). All samples were collected once for each participant between 2016 and 2017.
Data exclusions	The detailed list of inclusion and exclusion criteria is detailed by Eggers et al. (doi: 10.1136/bmjopen-2017-019450). In summary participants were excluded if they: 1) Reside in another location most of the time; 2) Are visitors to a household; and 3) Have a disclosed diagnosis of a mental disability.
Reproducibility	A subset of the Microbiome samples from this work was subjected to reproducibility testing in a recent study by Nikodemova et al. (doi: 10.3389/fcimb.2023.1165295). Attempts to repeat the experiments were successful.

Randomization	Survey participants were selected from a random sample of households using a three-stage, probability-based cluster sampling approach to generate a sampling frame. A detailed description of the randomization process is described by Malecki et al. (doi: 10.3389/fpubh.2022.818777)
Blinding	No blinding was used for this study.

Did the study involve field work? ☐ Yes ☒ No

Reporting for specific materials, systems and methods

We require information from authors about some types of materials, experimental systems and methods used in many studies. Here, indicate whether each material, system or method listed is relevant to your study. If you are not sure if a list item applies to your research, read the appropriate section before selecting a response.

Materials & experimental systems

n/a	Involved in the study	
☒	☐	Antibodies
☒	☐	Eukaryotic cell lines
☒	☐	Palaeontology and archaeology
☒	☐	Animals and other organisms
☒	☐	Clinical data
☒	☐	Dual use research of concern
☒	☐	Plants

Methods

n/a	Involved in the study	
☒	☐	ChIP-seq
☒	☐	Flow cytometry
☒	☐	MRI-based neuroimaging
